# Serine/threonine kinase 15 gene polymorphism and risk of digestive system cancers: A meta-analysis

**DOI:** 10.3892/etm.2014.2070

**Published:** 2014-11-14

**Authors:** JIANFEI LUO, RUICHENG YAN, LI ZOU

**Affiliations:** Department of Gastrointestinal Surgery, Renmin Hospital of Wuhan University, Wuhan, Hubei 430060, P.R. China

**Keywords:** serine/threonine kinase 15, polymorphism, digestive system cancers, meta-analysis

## Abstract

Previous studies have reported an association between the two coding polymorphisms (91T>A and 169G>A) of the serine/threonine kinase 15 (STK15) gene and the risk of digestive system cancers; however, the results are inconsistent. In the present study, a meta-analysis was carried out to assess the association between the two STK15 polymorphisms and the risk of digestive system cancers. Relevant studies were identified using PubMed, Web of Science, China National Knowledge Infrastructure, WanFang and VIP databases up to February 18, 2014. The pooled odds ratio (OR) with a 95% confidence interval (CI) was calculated using the fixed or random effects model. A total of 15 case-control studies from 14 publications were included. Of these, 15 studies concerned the 91T>A polymorphism and included 7,619 cases and 7,196 controls and four studies concerned the 161G>A polymorphism and included 826 cases and 713 controls. A significantly increased risk of digestive system cancers was observed for the 91T>A polymorphism (recessive model: OR, 1.19; 95% CI, 1.07–1.31). In subgroup analysis by ethnicity, a significant association was detected in Asian populations (recessive model: OR, 1.21; 95% CI, 1.08–1.36) but not in Caucasian and mixed populations. Stratification by tumor type indicated that the 91T>A polymorphism was associated with an increased risk of esophageal and colorectal cancers under the recessive model (OR, 1.19; 95% CI, 1.03–1.38; and OR, 1.24; 95% CI, 1.04–1.46; respectively); however, no significant association was observed between the 169G>A polymorphism and the risk of digestive system cancers in any of the genetic models. Furthermore, in subgroup analysis by ethnicity, similar results were observed in the Asian and Caucasian populations. The present meta-analysis demonstrated that the STK15 gene 91T>A polymorphism, but not the 169G>A polymorphism, may be a risk factor for digestive system cancers, particularly for esophageal and colorectal cancers.

## Introduction

Digestive system cancers, including esophageal, gastric, hepatocellular, bowel, pancreatic, gallbladder and anal cancers, are the most common types of cancer worldwide. There are an estimated 3.4 million new cases diagnosed worldwide each year and the mortality rates have increased over the past decade (1). Although the exact mechanism of carcinogenesis remains to be fully understood, accumulating evidence has confirmed that certain risk factors (such as dietary, ethnic and socioeconomic factors) and interactions between genetic and environmental factors may play important roles in the pathogenesis of these types of cancer (2,3).

Serine/threonine kinase 15 (STK15, also known as Aurora-A or AURKA) is a centrosome-localized serine/threonine kinase that is involved in cell cycle regulation, particularly the passage from G_2_ to M, through the formation of mitotic spindles (4). The STK15 gene, which consists of nine exons, is located on chromosome 20q13.2, a region frequently amplified and overexpressed in various types of human cancer (5). STK15 has been reported to be overexpressed in numerous types of malignancies, including colorectal and pancreatic cancers (6,7). Considerable evidence indicates that overexpression of the STK15 gene results in centrosome amplification, chromosomal instability, aneuploidy and transformation (8). Two non-synonymous polymorphisms, 91T>A (rs2273535) and 169G>A (rs1047972), have been identified in the STK15 gene. A thymine (T)/adenine (A) polymorphism located at nucleotide position 91 encodes a phenylalanine (Phe)-to-isoleucine (Ile) substitution at amino acid position 31. A guanine (G)/A polymorphism at nucleotide 169 encodes a valine (Val)-to-Ile substitution at amino acid position 57. The two polymorphisms are located within two conserved motifs in the N-terminus region of the STK15 gene (9). It has been revealed that the A allele of the 91T>A (31Ile>Phe) polymorphism is preferentially amplified and more potent than the T allele in leading to aneuploidy and transformation (8). Furthermore, the 169G>A (57Val>Ile) polymorphism was found to affect the kinase activity of aurora kinase A (10).

Studies have suggested the presence of an association between the two coding polymorphisms in the STK15 gene and an increased risk of digestive system cancers (10–23); however, the results have been inconsistent. The aim of the present study was therefore to conduct a meta-analysis to evaluate the association between the two STK15 polymorphisms and susceptibility to digestive system cancers.

## Materials and methods

### Search strategy

The electronic literature databases of PubMed, Web of Science, China National Knowledge Infrastructure (CNKI), WanFang and VIP were searched for all relevant articles. The last search update was February 18, 2014, using the search terms: ‘Serine/threonine kinase 15 or STK15 or Aurora-A or AURKA’ and ‘genetic polymorphism or polymorphisms or variant’ and ‘digestive system cancer or gastric cancer or colorectal cancer or hepatocellular carcinoma or pancreatic cancer or esophageal cancer’. The search was restricted to humans without language exclusions. Additional studies were identified by a manual search of the references from the original or review articles on this topic.

### Inclusion and exclusion criteria

Studies included in this meta-analysis were selected according to the following criteria: i) Studies that evaluated the association between the STK15 polymorphisms (91T>A or 169G>A) and digestive system cancers; ii) studies that had a case-control design; and iii) studies that had a detailed genotype frequency of cases and controls or that had presented sufficient data for this to be calculated from the article text. The major exclusion criteria were i) case-only studies, case reports and review articles; ii) studies without raw data of the STK15 genotype; and iii) repetitive publications.

### Data extraction

For each study, the following data were extracted independently by two investigators: The name of the first author, age and gender of the subjects, year of study publication, country of origin, ethnicity, source of controls, genotype methods, number of cases and controls, and the Hardy-Weinberg equilibrium (HWE) in the controls (P-value). The results were compared and disagreements were discussed among all authors and resolved with consensus.

### Statistical analysis

The HWE was evaluated for each study using an internet-based HWE calculator (http://ihg.gsf.de/cgi-bin/hw/hwa1.pl) (24). The risk of digestive system cancers associated with the STK15 polymorphisms was estimated for each study by the odds ratio (OR) and 95% confidence interval (CI). Four different ORs were calculated: The dominant model (variant homozygote + heterozygote versus wild-type homozygote), the recessive model (variant homozygote versus heterozygote + wild-type homozygote), heterozygote comparison (heterozygote versus wild-type homozygote) and homozygote comparison (variant homozygote versus wild-type homozygote). A χ^2^-test-based Q statistic test was performed to assess the heterogeneity between studies (25). The effect of heterogeneity was also quantified by the I^2^ test. When a significant Q test (P>0.05) or I^2^ value <50% indicated homogeneity across the studies, the fixed effects model was used (26); otherwise, the random effects model was used (27). Stratification analyses on ethnicity and tumor type were subsequently performed. Analysis of sensitivity was performed to evaluate the stability of the results. Finally, potential publication bias was investigated using Begg’s funnel plot and Egger’s regression test (28,29). P<0.05 was considered to indicate a statistically significant difference.

All analyses were performed using the Cochrane Collaboration RevMan 5.2 (The Nordic Cochrane Centre, The Cochrane Collaboration, Copenhagen, 2012) and STATA package version 12.0 (Stata Corporation, College Station, TX, USA).

## Results

### Study characteristics

**Figure 1:**
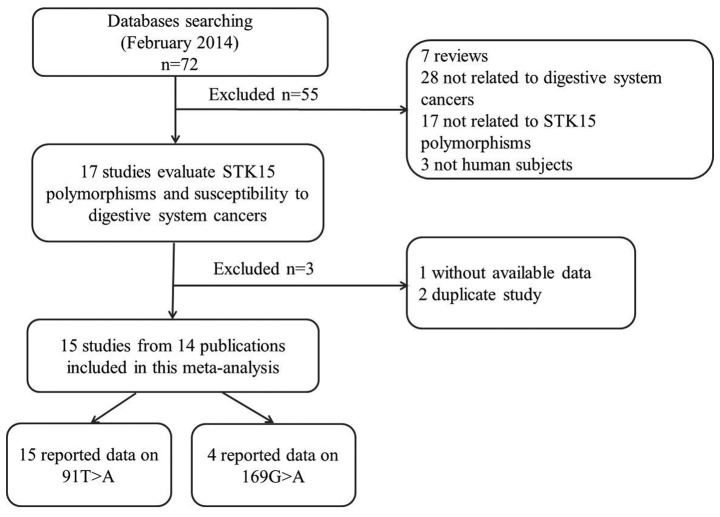
Flow chart showing the study selection procedure. STK15, serine/threonine kinase 15.

The search strategy retrieved 72 potentially relevant studies. According to the inclusion criteria, 14 studies (10–23) with full-text were included in the present meta-analysis and 58 studies were excluded. The flow chart of the study selection is summarized in Fig. 1. Since the study by Ewart-Toland *et al* (16) included two populations, these populations were treated separately in the current meta-analysis (Tables I and II); as such, there were 15 case-control studies from 14 publications with 7,619 cases and 7,196 controls concerning the 91T>A polymorphism and four studies with 826 cases and 713 controls concerning the 169G>A polymorphism. Of the 15 eligible studies, 10 studies (11,12,14,10,16–19,22) were written in English and five studies (13,15,20,21,23) in Chinese; nine studies (12,13,15,10,18–21,23) were conducted on Asian populations, five studies (11,14,16,17,22) on Caucasian populations and one study (16) on a mixed population. Four tumor types were addressed: Six studies (12,15,10,19–21) focused on esophageal cancer; six studies (14,16,17,22,23) on colorectal cancer; two studies (13,18) on gastric cancer and one study (11) on hepatocellular carcinoma. The distribution of genotypes among the controls was consistent with the HWE for all selected studies, with the exception of three (12,10,21).

### Quantitative data synthesis

**Figure 2:**
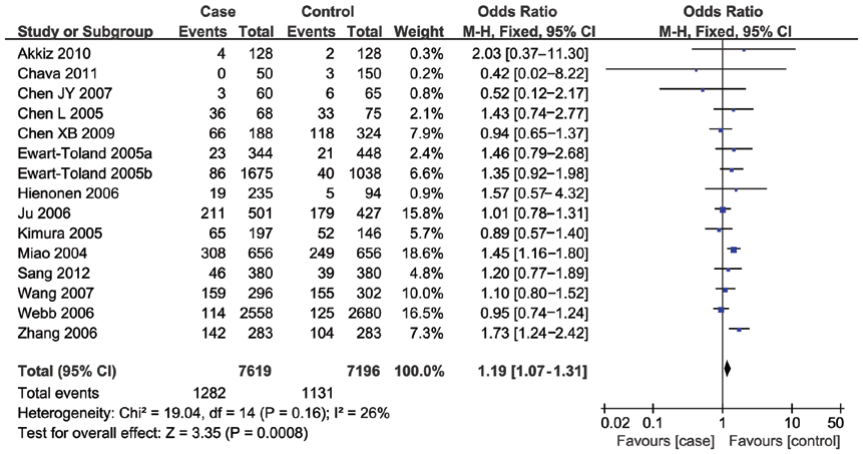
Forest plots for the association between the serine/threonine kinase 15 gene 91T>A polymorphism and digestive system cancers risk under a recessive model. CI, confidence interval.

Fifteen studies reported an association between the 91T>A polymorphism and susceptibility to digestive system cancers. Overall, a significantly increased risk was found under the recessive model (OR, 1.19; 95% CI, 1.07–1.31) (Fig. 2), while no notable associations were observed under the three other models (dominant model: OR, 1.02; 95% CI, 0.87–1.21; TA versus TT: OR, 0.97; 95% CI, 0.83–1.14; AA versus TT: OR, 1.12; 95% CI, 0.89–1.42).

In the subgroup analysis by ethnicity, a significant association was detected in the Asian population under the recessive model (OR, 1.21; 95% CI, 1.08–1.36) but under the other three models. No association was observed in the Caucasian or mixed populations.

Stratification by tumor type indicated that the 91T>A polymorphism was associated with an increased risk of esophageal and colorectal cancers under the recessive model (OR, 1.19; 95% CI, 1.03–1.38; and OR, 1.24; 95% CI, 1.04–1.46; respectively); however, no significant association was detected for gastric cancer. Only one study focused on hepatocellular cancer and the results showed that the STK15 91T>A polymorphism may be a genetic susceptibility factor for hepatocellular carcinoma (Table III).

Four studies reported an association between the 169G>A polymorphism and the risk of digestive system cancers. The combined results based on all the studies revealed no significant associations among the studies with any of the genetic models (dominant model: OR, 1.02; 95% CI, 0.82–1.28; recessive model: OR, 1.27; 95% CI, 0.25–6.49; GA versus GG: OR, 1.13; 95% CI, 0.90–1.43; AA versus GG: OR, 1.45; 95% CI, 0.29–7.22). In the subgroup analysis by ethnicity, similar results were demonstrated in the Asian and Caucasian populations (Table III).

### Heterogeneity and sensitivity analyses

Substantial heterogeneities were observed among the studies for the association between the risk of digestive system cancers and the 91T>A (dominant model: I^2^=68%, P<0.0001; TA versus TT: I^2^=63%, P=0.0005; AA versus TT: I^2^=58%, P=0.002) and 169G>A (recessive model: I^2^=58%, P=0.07; AA versus TT: I^2^=56%, P=0.08) STK19 polymorphisms. The source of the heterogeneity for the genetic model comparisons by ethnicity and tumor site was subsequently analyzed. For the 91T>A polymorphism, the heterogeneity was partially decreased or removed in colorectal and gastric cancers and Caucasian populations; however, significant heterogeneity remained for esophageal cancer and Asian populations. For the 169G>A polymorphism, the heterogeneity significantly decreased when the study by Kimura *et al* (10) was excluded from the analysis. A sensitivity analysis was performed to evaluate the stability of the results. Since the statistical significance of the results did not change when any single study was omitted, the stability of the results was confirmed.

### Publication bias

Begg’s funnel plot and Egger’s tests were used to address potential publication bias in the available literature. The shape of the funnel plots did not show any evidence of funnel plot asymmetry (data not shown). Egger’s test also demonstrated that there was no statistical significance in the evaluation of publication bias (dominant model, P=0.991; TA versus TT, P=0.721; AA versus TT, P=0.925; recessive model, P=0.835).

## Discussion

STK15, a member of the Aurora family, plays a vital role in bipolar mitotic spindle formation and regulates chromosome segregation in mammalian cells (30). It has been reported that STK15 is overexpressed in numerous types of cancer, including colorectal, pancreatic, breast and prostate (6,7,31,32). Although the mechanism remains unclear, it is believed that the polymorphism may partially affect STK15 expression and therefore modify its function. Ewart-Toland *et al* (8) suggested that the STK15 91T>A polymorphism (T→A) variant changed the activity of the STK15 box 1, leading to an inhibition of p53 binding and the decreased degradation of STK15. It was further suggested that the stabilized overexpression of STK15 led to centrosome amplification, improper cytokinesis, chromosomal instability and the promotion of tumorigenesis (8). To date, a number of studies have investigated the association between STK15 polymorphisms and the risk of cancers, particularly cancers of the digestive system (10–23); however, the results have been inconsistent. In a study from Turkey, Akkiz *et al* (11) reported that the STK15 91T>A polymorphism may be a genetic susceptibility factor for hepatocellular carcinoma. Similarly, Hienonen *et al* (17) observed that the STK15 91T>A polymorphism was a low penetrance colorectal cancer susceptibility factor in Finnish populations; however, Webb *et al* (22) suggested that there was no association between the polymorphism and colorectal cancer susceptibility based on their results. With regard to the 169G>A polymorphism, Ju *et al* (18) reported that the 169G>A polymorphism in the STK15 gene was associated with the progression of gastric cancer; however, in a study from China, Chen (13) failed to detect any association between the 169G>A polymorphism and the risk of gastric cancer.

Recently, two meta-analyses (33,34) evaluated the association between the STK15 91T>A polymorphism and risk of cancer, and reported that the STK15 91T>A polymorphism may be a risk factor for cancer. In comparison, the present study conducted a comprehensive literature search of different databases and included several additional studies. Furthermore, the association between the 169G>A polymorphism and the risk of digestive system cancers was explored. In the current meta-analysis, 15 studies were pooled to examine the association between the two STK15 polymorphisms and risk of digestive system cancers. The results demonstrated that there was a significant association between the STK15 91T>A polymorphism and the risk of digestive system cancers.

In the subgroup analysis by ethnicity, there was a significant association in Asian descent, but not in Caucasian and mixed populations. Different genetic backgrounds and environmental exposures among the different ethnic groups may contribute to this discrepancy (35). When stratified by tumor type, the 91T>A polymorphism was associated with an increased risk of esophageal and colorectal cancers, but not gastric cancer. Only one study focused on hepatocellular carcinoma and the results revealed that the STK15 91T>A polymorphism may be a genetic susceptibility factor for hepatocellular carcinoma; however, since only a few studies on gastric cancer and hepatocellular carcinoma were included, these results should be interpreted with caution, and further studies are required.

No significant association was found between the 169G>A polymorphism and the risk of digestive system cancers in any of the genetic models. When stratified according to ethnicity, similar results were observed in Asian and Caucasian populations. This lack of association may have been due to the limited literature (only four studies) in the present meta-analysis. The conclusions should therefore be considered sensibly. Furthermore, cancer is a multi-factorial disease that results from complex interactions between a number of environmental and genetic factors (gene-gene or gene-environment). Not all of the studies included, however, analyzed the same environmental or genetic factors and, due to lack of individual data in the present review, more detailed analyses, such as analyses of joint effects with other risk factors or gene-gene or gene-environment interactions, were not able to be performed.

Heterogeneity is a potential problem when interpreting the results of all meta-analyses (36). In the current meta-analysis, heterogeneity was observed in the overall comparison for certain genetic models. When stratified by ethnicity and tumor site, the heterogeneity was partially decreased or removed in colorectal and gastric cancers and Caucasian populations; however, heterogeneity remained for esophageal cancer and Asian populations. For the 169G>A polymorphism, the heterogeneity significantly decreased when the study by Kimura *et al* (10) was excluded from analysis. These results suggest that the ethnic difference, different tumor types and particular study type may be the source of heterogeneity in the present meta-analysis. When sensitivity analyses were conducted by successively excluding one study, the estimated pooled OR changed little, strengthening the results from the meta-analysis. Furthermore, no publication bias was observed, highlighting the possibility of true results.

The current meta-analysis has limitations that require acknowledgement. Firstly, due to incomplete raw data or publication limitations, certain relevant studies were unable to be included in the present analysis. Secondly, the results were based on unadjusted estimates, which may cause serious confounding bias. Thirdly, the data from the European populations were relatively small and significant heterogeneity was observed in certain models, which may have resulted in failure to confirm marginal associations.

In conclusion, the present meta-analysis suggests that the STK15 gene 91T>A polymorphism, but not the 169G>A polymorphism, may be a risk factor for digestive system cancers, particularly for esophageal and colorectal cancers.

## Figures and Tables

**Table I:** Characteristics of studies included in the meta-analysis.

A, Studies on the 91T>A polymorphism						

First author (ref.)	Year	Country	Ethnicity	Tumor type	Source of controls	Genotype methods
Akkiz (11)	2010	Turkey	Caucasian	Hepatocellular	HB	PCR-RFLP
Chava (12)	2011	India	Asian	Esophageal	NR	PCR
Chen L (13)	2005	China	Asian	Gastric	HB	PCR-RFLP
Chen JY (14)	2007	USA	Caucasian	Colorectal	HB	Direct sequencing
Chen XB (15)	2009	China	Asian	Esophageal	PB	PCR-RFLP
Ewart-Toland (16)	2005a	USA	Mixed	Colorectal	PB	PCR-RFLP
Ewart-Toland (16)	2005b	Scotland	Caucasian	Colorectal	PB	PCR-RFLP
Hienonen (17)	2006	Finland	Caucasian	Colorectal	PB	Direct sequencing
Ju (18)	2006	South Korea	Asian	Gastric	HB	Mass ARRAY
Kimura (10)	2005	Japan	Asian	Esophageal	HB	PCR
Miao (19)	2004	China	Asian	Esophageal	PB	PCR-RFLP
Sang (20)	2012	China	Asian	Esophageal	HB	MALDI-TOF MS
Wang (21)	2007	China	Asian	Esophageal	PB	PCR-RFLP
Webb (22)	2006	UK	Caucasian	Colorectal	PB	Illuminasentric bead array
Zhang (23)	2006	China	Asian	Colorectal	PB	PCR-RFLP

B, Studies on the 169G>A polymorphism						

First author (ref.)	Year	Country	Ethnicity	Tumor type	Source of controls	Genotype methods

Chen L (12)	2005	China	Asian	Gastric	HB	PCR-RFLP
Chen JY (13)	2007	USA	Caucasian	Colorectal	HB	Direct sequencing
Ju (17)	2006	South Korea	Asian	Gastric	HB	Mass ARRAY
Kimura (18)	2005	Japan	Asian	Esophageal	HB	PCR

NR, not reported; HB, hospital-based; PB, population-based; MALDI-TOF MS, matrix-assisted laser desorption/ionization time of flight mass spectrometry; PCR-RFLP, polymerase chain reaction-restriction fragment length polymorphism; ref., reference.

**Table II:** Patient data for studies included in the meta-analysis.

A, Studies on the 91T>A polymorphism									

	Age (years)		Gender (male/female)		Genotype (case/control)				
				
First author (ref.)	Case	Control	Case	Control	Total	WT Ho (TT)	Ht (TA)	VR Ho (AA)	P-value_HWE_
Akkiz (11)	58 (20–81)^a^	58 (20–81)^a^	106/22	106/22	128/128	77/99	47/27	4/2	0.919
Chava (12)	56.03	NR	NR	NR	50/150	22/81	28/66	0/3	0.012
Chen L (13)	49.04±12.89^b^	51.66±16.12^b^	45/23	38/37	68/75	5/10	27/32	36/33	0.615
Chen JY (14)	43.0±12.7^b^	44.8±12.0^b^	36/24	27/38	60/65	44/38	13/21	3/6	0.236
Chen XB (15)	NR	NR	178/10	307/17	188/324	43/38	79/168	66/118	0.060
Ewart-Toland (16)	NR	NR	NR	NR	344/448	200/279	121/148	23/21	0.809
Ewart-Toland (16)	NR	NR	NR	NR	1675/1038	1031/630	558/368	86/40	0.126
Hienonen (17)	68 (32–90)^a^	NR	109/126	NR	235/94	122/46	94/43	19/5	0.208
Ju (18)	57.7±12.6^b^	52.4±8.7^b^	339/162	289/138	501/427	75/58	215/190	211/179	0.504
Kimura (10)	NR	NR	NR	NR	197/146	29/12	103/82	65/52	0.010
Miao (19)	58.3±9.6^b^	57.5±9.5^b^	460/196	443/213	656/656	58/91	290/316	308/249	0.560
Sang (20)	NR	NR	NR	NR	380/380	173/153	161/188	46/39	0.089
Wang (21)	59.8±9.7^b^	58.8±7.9^b^	202/94	202/100	296/302	34/36	103/111	159/155	0.026
Webb (22)	61±11.4^b^	59±10.9^b^	1471/1087	836/1844	2558/2680	1564/1667	880/888	114/125	0.628
Zhang (23)	57.0±11.0^b^	57.4±9.6^b^	171/112	170/113	283/283	30/42	111/137	142/104	0.775

B, Studies on the 169G>A polymorphism									

	Age (years)		Gender (male/female)		Genotype (case/control)				
				
First author (ref.)	Case	Control	Case	Control	Total	WT Ho (GG)	Ht (GA)	VR Ho (AA)	P-value_HWE_

Chen L (13)	49.04±12.89^b^	51.66±16.12^b^	45/23	38/37	68/75	49/61	19/11	0/3	0.019
Chen JY (14)	43.0±12.7^b^	44.8±12.0^b^	36/24	27/38	60/65	39/43	20/20	1/2	0.859
Ju (18)	57.7±12.6^b^	52.4±8.7^b^	339/162	289/138	501/427	387/414	100/104	14/9	0.409
Kimura (10)	NR	NR	NR	NR	197/146	118/99	65/47	14/0	0.020

a Presented as the mean (range);

b presented as the mean ± standard deviation;

HWE, Hardy-Weinberg equilibrium; NR, not reported; Ht, heterozygote; VR Ho, variant homozygote; WT Ho, wild-type homozygote; ref., reference.

**Table III:** Summary of the ORs of the serine/threonine kinase 15 polymorphisms and risk of digestive system cancers.

A, Studies on the 91T>A polymorphism													

		Dominant model			Recessive model			Ht versus WT Ho			VR versus WT Ho		
					
Variables	N^a^	OR (95% CI)	P-value^b^	I^2^	OR (95% CI)	P-value^b^	I^2^	OR (95% CI)	P-value^b^	I^2^	OR (95% CI)	P-value^b^	I^2^
Total	15	1.02 (0.87–1.21)	<0.0001	68	1.19 (1.07–1.31)	0.16	26	0.97 (0.83–1.14)	0.0005	63	1.12 (0.89–1.42)	0.002	58
Ethnicity													
Asian	9	0.99 (0.73–1.35)	0.0002	73	1.21 (1.08–1.36)	0.10	40	0.92 (0.69–1.22)	0.003	66	1.07 (0.74–1.55)	0.0005	71
Caucasian	5	1.04 (0.83–1.29)	0.01	68	1.08 (0.88–1.32)	0.37	6	1.02 (0.81–1.28)	0.01	68	1.08 (0.88–1.33)	0.36	9
Mixed	1	1.19 (0.89–1.58)	NA	NA	1.46 (0.79–2.68)	NA	NA	1.14 (0.84–1.54)	NA	NA	1.12 (0.99–1.28)	NA	NA
Tumor type													
Esophageal	6	0.90 (0.59–1.37)	0.0001	80	1.19 (1.03–1.38)	0.24	26	0.85 (0.57–1.28)	0.0007	77	0.91 (0.55–1.53)	0.0006	77
Colorectal	6	1.03 (0.95–1.12)	0.20	31	1.24 (1.04–1.46)	0.08	49	1.01 (0.93–1.10)	0.37	8	1.18 (0.98–1.42)	0.15	39
Gastric	2	0.97 (0.68–1.37)	0.20	39	1.06 (0.83–1.35)	0.33	0	0.94 (0.65–1.36)	0.30	5	1.00 (0.69–1.45)	0.17	48
Hepatocellular	1	2.26 (1.31–3.90)	NA	NA	2.03 (0.37–11.30)	NA	NA	2.24 (1.28–3.92)	NA	NA	2.57 (0.46–14.41)	NA	NA

B, Studies on the 169G>A polymorphism													

		Dominant model			Recessive model			Ht versus WT Ho			VR versus WT Ho		
					
Variables	N^a^	OR (95% CI)	P-value^b^	I^2^	OR (95% CI)	P-value^b^	I^2^	OR (95% CI)	P-value^b^	I^2^	OR (95% CI)	P-value^b^	I^2^

Total	4	1.02 (0.82–1.28)	0.13	47	1.27 (0.25–6.49)	0.07	58	1.13 (0.90–1.43)	0.45	0	1.45 (0.29–7.22)	0.08	56
Ethnicity													
Asian	3	0.98 (0.77–1.24)	0.14	49	2.04 (0.32–13.00)	0.07	62	1.07 (0.84–1.36)	0.91	0	2.29 (0.38–13.69)	0.09	59
Caucasian	1	1.69 (0.77–3.71)	NA	NA	0.15 (0.01–2.98)	NA	NA	2.15 (0.94–4.94)	NA	NA	0.18 (0.01–3.52)	NA	NA

a Number of comparisons;

b test for heterogeneity.

NA, not applicable; CI, confidence interval; OR, odds ratio; Ht, heterozygote; WT Ho, wild-type homozygote; VR, variant.
